# COVID-19 and Infant Hospitalizations for Seasonal Respiratory Virus Infections, New Zealand, 2020

**DOI:** 10.3201/eid2702.204041

**Published:** 2021-02

**Authors:** Adrian Trenholme, Rachel Webb, Shirley Lawrence, Sharon Arrol, Susan Taylor, Shanthi Ameratunga, Catherine A. Byrnes

**Affiliations:** Kidz First Children’s Hospital, Auckland, New Zealand (A. Trenholme, R. Webb, S. Lawrence);; University of Auckland, Auckland (A. Trenholme, R. Webb, C.A. Byrnes);; Department of Health Informatics, Auckland (R. Webb, S. Arrol);; Middlemore Hospital, Auckland (S. Arrol, S. Taylor. S. Ameratunga);; Population Health Directorate, Auckland (S. Ameratunga);; Starship Children’s Health, Auckland (C.A. Byrnes)

**Keywords:** 2019 novel coronavirus disease, coronavirus disease, COVID-19, severe acute respiratory syndrome coronavirus 2, SARS-CoV-2, viruses, respiratory infections, zoonoses, seasonal, influenza, respiratory syncytial virus, lower respiratory tract infection, hospitalizations, infants, South Auckland, New Zealand

## Abstract

In March 2020, a national elimination strategy for coronavirus disease was introduced in New Zealand. Since then, hospitalizations for lower respiratory tract infection among infants <2 years of age and cases of respiratory syncytial or influenza virus infection have dramatically decreased. These findings indicate additional benefits of coronavirus disease control strategies.

In New Zealand, the incidence of hospitalization of infants with lower respiratory tract infection (LRTI) is high. LRTIs disproportionately affect Māori and Pacific Islander children and are predominantly caused by respiratory syncytial virus (RSV) (*1*).

The first case of coronavirus disease (COVID-19) in New Zealand was identified on February 28, 2020. Subsequently, the government pursued an elimination strategy, commencing with a national lockdown on March 25, along with strict international border controls, mandatory 14-day isolation of all international arriving passengers, intensive community testing, school closures, and contact tracing. This strategy seems to have largely succeeded, although recent small clusters of cases in the Auckland region demonstrate continuing vulnerabilities (*2*).

Kidz First Children’s Hospital serves an urban population of ≈550,000 persons in South Auckland, where 50% of infants are of Māori or Pacific Islander ethnicity. Since 2007, clinicians have performed nasopharyngeal sampling for respiratory virus PCR when clinically indicated and have participated in the SHIVERS (Southern Hemisphere Influenza Vaccine Effectiveness Surveillance) program of multiplex PCR virus surveillance (*2*). Since March 2020, additional COVID-19 PCR testing has been routinely performed for hospitalized children with respiratory illness. Influenza vaccine, although recommended for pregnant women and high-risk infants and children, is not routinely administered. During winter–spring 2019, a large measles outbreak occurred in Auckland and hospitalizations increased. From 2016 through 2019, a randomized clinical trial of RSV vaccine for pregnant women was conducted with 152 South Auckland mother–infant pairs (*3*).

After COVID-19 lockdown measures were implemented, we observed a marked reduction in hospitalizations of infants for respiratory illness at Kidz First Hospital; the reduction was sustained after gradual easing of the national lockdown beginning on April 27, 2020. To confirm the decrease, we examined respiratory viral PCR test results and infant LRTI hospitalization data from January 1, 2015, through August 31, 2020. We reviewed clinical and laboratory records of infants <2 years of age hospitalized for >3 hours during that time for LRTI (codes J22, A37, J47, J10.0 J10.1 J11.1, J12–16, J20, J21, and J18 from the International Classification of Diseases, 10th Revision). All specimens submitted by a clinician for respiratory viral PCR testing were identified. Re-admissions and duplicate tests were not excluded from this dataset.

Annual numbers of hospitalizations for LRTI during 2015–2019 varied from 1,486 to 2,046. A characteristic winter peak in hospitalizations occurred during July and August; however, from January 1 through August 31, 2020, only 268 admissions were reported, with no winter peak observed (Figure). Numbers of clinician-directed PCR tests performed during March 1–August 31 during the 6-year study period are similar except for increased testing in 2019 during the major measles outbreak (Table). Since March 2020, the numbers of hospitalizations associated with a positive PCR result for RSV (n = 2) and influenza (n = 1) have plummeted; however, hospitalizations for adenovirus and rhinovirus/enterovirus (positive by PCR) have persisted at levels similar to previous years. No hospitalized children have received positive COVID-19 test results.

**Figure:**
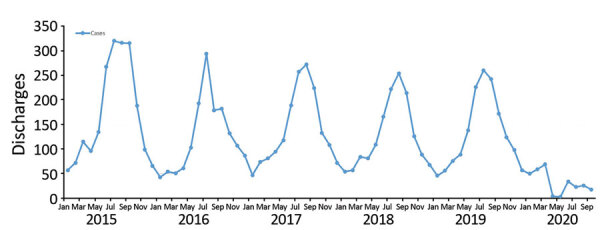
Hospital discharges among children <2 years of age with lower respiratory tract infection, South Auckland, New Zealand, 2015–2019**.**

**Table:** Data for children <2 years of age hospitalized for LRTI, South Auckland, New Zealand, March 1–August 31, 2015–2020*

Variable	2015	2016	2017	2018	2019	2020
Total hospitalizations for LRTI	1,249	881	1,012	916	1,031	159
Total PCR tests for LRTI viruses	7,259	6,642	8,876	7,676	14,881	6,735
Positive PCR results						
RSV	214	224	317	204	388	2
Influenza A	28	16	56	53	85	1
Influenza B	11	6	11	1	97	0
Rhinovirus/enterovirus	285	274	378	283	495	252
Adenovirus	106	26	83	66	72	41

*LRTI, lower respiratory tract infection; RSV, respiratory syncytial virus.

The New Zealand COVID-19 elimination strategy seems to have halted transmission of seasonal RSV and influenza virus to infants in South Auckland; similar findings have been reported for other populations around the world, focused mainly on influenza reductions (*4*–*8*). The most likely influence on the virtual absence of RSV and influenza disease affecting infants (during what would usually be the peak winter season in New Zealand) is international border controls, including mandatory 14-day isolation of arriving passengers, limiting seasonal virus ingress to the country, although physical distancing and hygiene measures undoubtedly play a part. This hypothesis is further supported by the persistence of rhinovirus/enterovirus infections and lack of rebound of RSV and influenza infections when lockdown measures were gradually eased from late April on. The persistence of disease burdens from viruses that circulate all year suggests that although border controls have prevented entry of the seasonal viruses into the population, community preventive measures have had a more limited effect on the transmission of regional endemic viruses that cause infant hospitalizations.

Our findings are supported by the informative comparison of data across 6 years, during which time the clinician-directed investigation of infants with respiratory infections has remained consistent. Although these preliminary single-center findings need confirmation over a complete year and with national-level surveillance data, they closely align with emerging reports from Alaska, Australia, and Finland (*5*,*8*,*9*).

The current global situation emphasizes the need for ongoing comprehensive respiratory virus surveillance in vulnerable populations, as demonstrated by the unexpected benefit seen locally for Māori and Pacific Islander infants. As the Northern Hemisphere winter approaches, the population-level benefits of substantially reduced RSV and influenza burden may usefully inform policy makers about the merits of different COVID-19 control strategies (*10*).
